# Design of RGDS Peptide-Immobilized Self-Assembling β-Strand Peptide from Barnacle Protein

**DOI:** 10.3390/ijms22031240

**Published:** 2021-01-27

**Authors:** Daisuke Fujii, Kento Takase, Ami Takagi, Kei Kamino, Yoshiaki Hirano

**Affiliations:** 1 Faculty of Chemistry, Materials and Bioengineering, Kansai University, Suita, Osaka 564-8680, Japan; k684451@kansai-u.ac.jp (D.F.); grgdspsspep5885@gmail.com (K.T.); k813045@kansai-u.ac.jp (A.T.); 2 National Institute of Technology and Evaluation, Kisarazu, Ciba 292-0818, Japan; kamino-kei@nite.go.jp; 3 Organization for Research and Development of Innovative Science and Technology, Kansai University, Suita, Osaka 564-8680, Japan

**Keywords:** barnacle adhesive proteins, self-assembling peptide, β-sheet peptide, RGD, peptide hydrogel

## Abstract

We designed three types of RGD-containing barnacle adhesive proteins using self-assembling peptides. In the present study, three types of RGD-containing peptides were synthesized by solid-phase peptide synthesis, and the secondary structures of these peptides were analyzed by CD and FT-IR spectroscopy. The mechanical properties of peptide hydrogels were characterized by a rheometer. We discuss the correlation between the peptide conformation, and cell attachment and cell spreading activity from the viewpoint of developing effective tissue engineering scaffolds. We created a peptide-coated cell culture substrate by coating peptides on a polystyrene plate. They significantly facilitated cell adhesion and spreading compared to a non-coated substrate. When the RGDS sequence was modified at N- or C-terminal of R-Y, it was found that the self-assembling ability was dependent on the strongly affects hydrogel formation and cell adhesion caused by its secondary structure.

## 1. Introduction

In recent years, the self-assembly of peptides using β-sheet structure has been actively conducted in the fields of functional hydrogels, nanomaterials, and biomaterials [1,2,3,4,5,6,7,8,9]. In biomaterials, the use of chemical compounds is popular in various fields such as organic polymers, artificial proteins, and sugars. Peptides are attracting attention because they can be synthesized by sequentially condensing amino acids and have the property of being absorbed by metabolism even when they are embedded in the body. The famous peptide used in biomaterials in vivo and in vitro is a self-assembling peptide such as β-strand peptide [10,11,12], β-hairpin peptide [13,14,15,16,17,18,19,20,21,22,23], and small molecule peptide [24,25,26,27,28], and they form a self-assembling nanofiber network and a hydrogel by adding a salt to the aqueous peptide solution [29,30,31,32,33]. This peptide hydrogel has a highly hydrous and porous structure similar to that of the natural extracellular matrix. Due to these properties, a self-assembling peptide is used as a scaffold material for cell culture, and research is being actively conducted in each field. If, however, a self-assembling peptide is used as it is, it may not be possible to attain sufficient activity. Therefore, some modifications are called for, such as introducing a bioactive peptide only to its C-terminal to achieve the desired activity [34,35,36,37,38].

However, there is no research examining the modification position of the bioactive motif for evaluation of possible scaffold materials. In general, in the β-sheet structure, the formation position of intermolecular hydrogen bonds and intramolecular hydrogen bonds of peptides is determined by the amino acid sequence and the number of amino acid residues. When a bioactive motif such as RGDS is modified for a self-assembled peptide, the physical properties also alter because the peptide sequence changes depending on how the bioactive motif is modified. When the position of the intermolecular hydrogen bond changes, the conformation and physical properties of peptide is changed. It is considered that this also changes the physical properties of the peptide hydrogel. From the above, when designing a new peptide sequence, it is necessary to systematically investigate the sequence and physical properties of the peptide. Therefore, in this study, we investigated the different effects on the peptide’s physical properties by changing the modification position of the bioactive motif on the self-assembling peptide [39,40,41,42].

The peptide sequence in the barnacle-derived protein was selected and used as the self-assembling peptide sequence. Barnacles are crustacean organisms that adhere to seaside rocks, ship bottoms, and various interfaces in seawater [43,44,45]. Multiple proteins are involved in this adhesion mechanism. These barnacle proteins include those that can be absorbed into a substrate, suppress proteolysis by microorganisms, and function as bulk substances. Among them, there is a protein called cp-52k that functions as a bulk substance [46].

This protein exists in a solution state in the body of barnacles, but when it goes out of the body, a part of the protein sequence is transformed to a β-sheet structure by salt in seawater. The conformational changes of these peptides form from sol to gel. The sequence of cp-52k that most sensitively responds to salts and stably forms a β-sheet structure is Arg-Arg-Lys-Ser-Tyr-Ser-Gly-Ile-Leu-Gly-Asp-Leu-Ile-Gln-Ala-Val-Ile-Arg-Tyr-Tyr (R-Y) [47,48]. However, since the R-Y sequence alone does not have cell adhesion, even when a hydrogel is produced by self-assembly, it cannot be expected to be a functional scaffold material. Therefore, the R-Y sequence was modified with the RGDS cell adhesion peptide derived from fibronectin.

In this study, we designed a peptide of which the self-assembling peptide R-Y was modified with the cell adhesion peptide RGDS [39]. Three types of molecules were designed: R-Y-RGDS with RGDS modified at the C-terminus of R-Y, RGDS-R-Y with RGDS modified at the N-terminus of R-Y, and R-RGD-Y with RGD introduced into the sequence of R-Y. R-RGD-Y was replaced leucine with arginine in the center of R-Y; -LGD- sequence to -RGD-. Then, the function of each peptide was evaluated, and the correlations among self-assembling ability, physical properties, and biological activity resulting from different modification positions of RGDS were examined. Specifically, we compared and examined elements that are thought to be related to physical properties and biological activity, such as the ability to form β-sheet structures, the strength of hydrogels, and the ability to adhere to cells.

## 2. Results and Discussion

### 2.1. Peptide Synthesis

Four types of peptides in Table 1 were synthesized manually by solid phase peptide synthesis. All peptides were characterized by HPLC, MALDI-TOF-MS and amino-acid analysis. All peptides were purified by RT- HPLC to a single peak. The results of MALDI-TOF-MS spectra m/Z of [M+1]^+^, [M+Na]^+^, and [M+K]^+^ were shown in Table 2. These results suggest that 4 kinds of peptides were synthesized as intended in the molecular design.

### 2.2. Secondary Structure Analysis by Infrared Absorption (IR) Spectrum

The R-Y, R-Y-RGDS, RGDS-R-Y, and R-RGD-Y show absorption from the amide I bond between 1600 cm^−1^ and 1700 cm^−1^, and in particular the β-sheet structure was found near 1630 cm^−1^ (Supplementary Figure S1). The secondary structure calculated using multivariate analysis from waveforms from IR spectra from the R-Y, R-Y-RGDS, RGDS-R-Y, and R-RGD-Y are shown in Table 2. It shows that all peptides contain a β-sheet and β-turn structure. From these results, it was clarified that the RGDS modification to the N-terminal of the R-Y and the change of the LGD sequence in the R-Y to RGD do not significantly affect the amount of β-sheet structure. On the other hand, R-Y-RGDS contains 50% β-sheet structure, which is less than R-Y, RGDS-R-Y, and R-RGD-Y type peptides. Since the intermolecular interaction is large in solid state peptide samples, it seems that the β-sheet structure is strongly observed by FT-IR in R-Y-RGDS. This result clarified that RGDS modification to the C-terminal of the R-Y inhibits its β-sheet structure formation ability.

### 2.3. Secondary Structure Analysis by Circular Dichroism (CD) Spectrum

**Figure 1:**
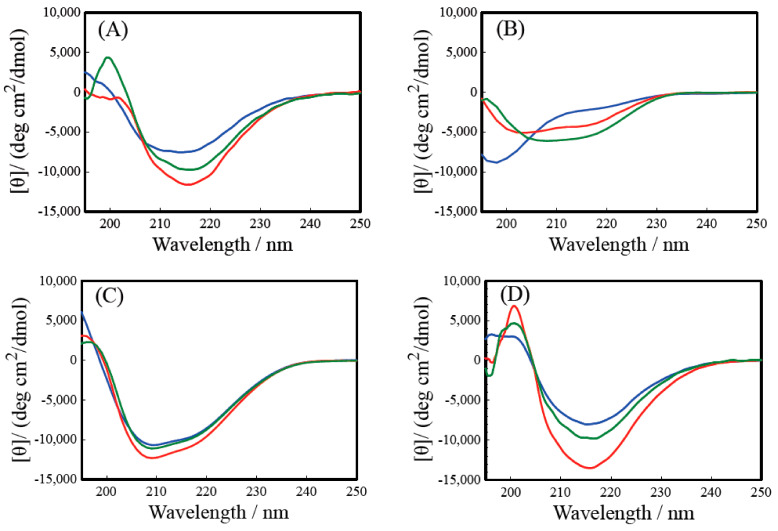
CD spectra of synthetic R-Y derivative peptide. (**A**) R-Y, (**B**) R-Y-RGDS, (**C**) RGDS-R-Y, (**D**) R-RGD-Y. H_2_O: Blue line, 0.06 M NaCl; Green line, 0.10 M NaCl; Red line.

The CD spectra of R-Y, R-Y-RGDS, RGDS-R-Y, and R-RGD-Y are shown in Figure 1. All samples’ HT values in the CD measurement were less than 500 in 200–250 nm. In 195–200 nm, HT values were less than 600. Figure 1A confirms a negative Cotton effect around 215 nm caused by the β-sheet structure for all salt concentrations (pH = 7.0–7.4). It was observed that the minimum value decreases as the salt concentration increases. From this result, it was shown that the R-Y forms a β-sheet structure in an aqueous solution and a NaCl aqueous solution, and that the formation of a β-sheet structure is dependent on the salt concentration. When the R-Y forms a β-sheet structure in an aqueous solution, the positive charges present in the side chains of arginine and lysine have an electrostatic intermolecular interaction; thus, intermolecular force is repelled. So, the chloride ion was added to the solution, blocking the positive charge and suppressing the repulsion. It was shown that R-RGD-Y forms a β-sheet structure in aqueous solution and NaCl solution, and that β-sheet structure formation is facilitated as the salt concentration increases. This indicates that even when LGD in the R-Y sequence is changed to RGD, the NaCl concentration is dependent on the β-sheet structure of R-Y (Figure 1D). By changing LGD to RGD, the positions of intermolecular and intramolecular hydrogen bond in peptides were changed and affected the conformation. Also, the RGDS sequence is generally known to form a turn structure at the Gly site; despite these, the results show that this sequence does not change the formation of a secondary structure. This supports that the R-Y is more likely to form a β-turn structure than a general β-sheet structure. It can be inferred that R-RGD-Y and R-Y have very similar structures. From Figure 1C, it can be confirmed that the RGDS-R-Y forms the β-sheet structure at all salt concentrations, and that the negative Cotton effect (205 nm) become greater as the NaCl concentration higher. This result shows that the RGDS-RY forms a β-sheet structure in an aqueous solution and a salt aqueous solution, and that the formation of a β-sheet structure is facilitated as the NaCl concentration increases. It was possible that peptide solution was coexistent β-sheet structure conformer and small quantity of α-helix conformer. So, in solid state peptide, the intermolecular interaction was larger than solution state, however CD measurement was carried out in dilute solution. Therefore, there are some difference in the results of secondary structure of CD and IR. Comparing the R-Y and RGDS-R-Y, the wavelength showing the negative Cotton effect is shifted to a shorter wavelength, which is observed in the spectrum of a general β-sheet structure. On the other hand, Figure 1B shows that the R-Y-RGDS forms a random coil structure in the aqueous solution and a weak β-sheet structure is formed in the NaCl solution. It can be confirmed that the negative Cotton effect around 205 to 215 nm becomes greater, indicating the dependency on the NaCl concentration. This result clarified that the β-sheet structure forming ability suffered when the RGDS was modified at the C-terminal of the R-Y.

From the above results of the CD and IR spectra, it was found that the RGDS modification to the N-terminal of R-Y and the change of LGD in the R-Y sequence to RGD do not significantly affect the β-sheet structure forming ability. With the RGDS modified at the N-terminal, the amount of the β-sheet structure was greater than the R-Y. Thus, with the RGDS modification to the C-terminal of the R-Y, the β-sheet formation was inhibited more compared to the R-Y. It is concluded that the likelihood of β-sheet structure formation influences the self-assembling mechanism of the peptides’ interaction.

### 2.4. Preparation of Peptide Hydrogel and Evaluation of Physical Properties

**Figure 2:**
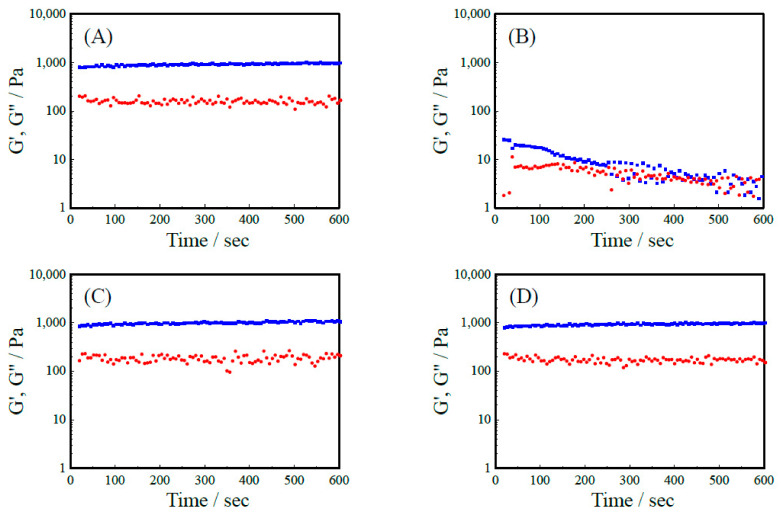
Results of rheology measurement for 2 wt% hydrogel. (**A**) R-Y, (**B**) R-Y-RGDS, (**C**) RGDS-R-Y, (**D**) R-RGD-Y. Blue dot; G’, Rad dot: G’.

Figure 2 shows the elastic modulus (G’) and the loss elastic modulus (G’’) of R-Y, R-Y-RGDS, RGDS-R-Y, and R-RGD-Y peptide hydrogel. It was revealed that G’ exceeded G” in all 3.0 wt% peptide hydrogels. These results suggest that a peptide hydrogel was formed. The G’ of R-RGD-Y showed a higher value compared to the R-Y, but the G’ of the RGDS-R-Y showed a value about 700 Pa smaller than that of the R-Y. From the secondary structure analysis of CD and IR, it can be considered that the RGDS-R-Y contains more β-sheet structure than the R-Y. These results imply that the mechanical properties of the peptide hydrogel having the backbone of R-Y are enhanced when it contains more β-sheet structures. RGDS-R-Y and R-RGD-Y also formed peptide hydrogels because their G’ exceeded G” at 2 wt%. On the other hand, 1 wt% R-Y-RGDS solution took a sol formation, and 2 wt% and 3 wt% formed a hydrogel close to the sol (Table 3). This correlates with the results of secondary structure analysis of CD and IR, and the smallest G’ of R-Y-RGDS, which has the weakest ability to form β-sheet structures. It can be inferred that there is a correlation between the β-sheet structure forming ability and the mechanical strength of the hydrogel. The viscoelasticity of a peptide hydrogel depends on the concentration of the peptide.

However, it was difficult for the R-Y-RGDS to form a hydrogel. It is considered that self-assembly did not occur because the secondary structure of the peptide did not form a β-sheet structure. In addition, it can be considered that the RGDS modified at the N terminal of the R-Y inhibited the formation of β-sheet structure (Table 3).

### 2.5. Observation of Microstructure of Peptide Hydrogel with Scanning Electron Microscope (SEM)

**Figure 3:**
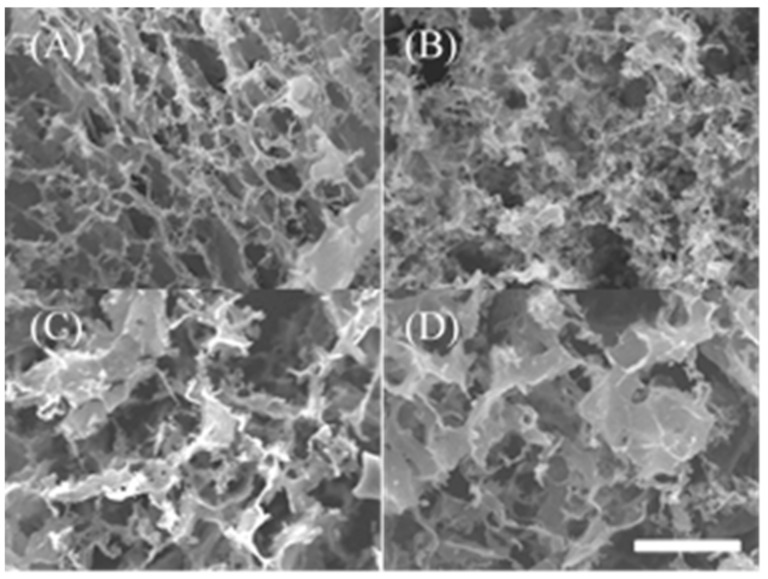
SEM image of 2wt% hydrogel. (×2000) (Scale bar: 5 micro m). (**A**) R-Y, (**B**) R-Y-RGDS, (**C**) RGDS-R-Y, (**D**) R-RGD-Y.

SEM images of peptide hydrogels of 2 wt% R-Y, R-Y-RGDS, RGDS-R-Y, and R-RGD-Y are shown in Figure 3. Figure 3A,B show that the 2 wt% RY and R-Y-RGDS peptide hydrogel formed nanofibers and constructed a network structure. The pores of R-Y-RGDS were dense compared with SEM images of RGDS-R-Y and R-RGD-Y. Figure 3C,D also formed a porous structure, but the shape was on the scale and the morphology was different from that of A and B. According to the secondary structure analysis, the modification position of RGDS has the β-sheet structure forming ability and the secondary structure is influential. 

### 2.6. Cell Culture on a Peptide-Immobilized Substrate

**Figure 4:**
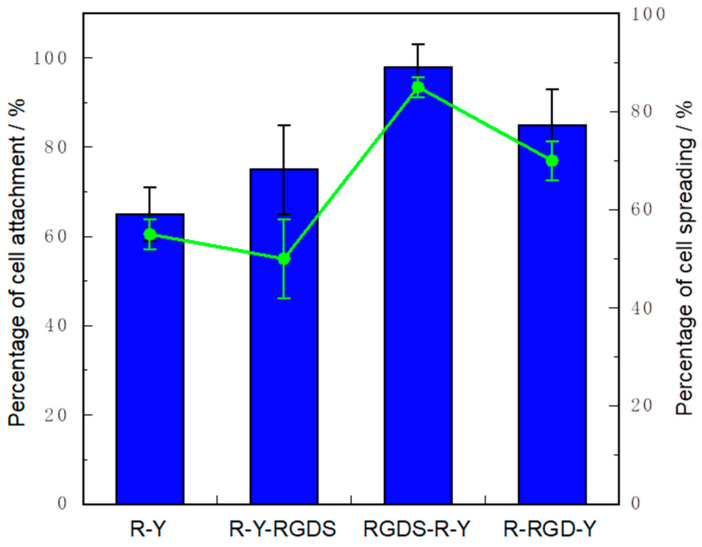
Result of cell attachment and cell spreading activity of R-Y derivative peptide modified onto cell culture plate. Incubation time; 6 h. * *p* < 0.05 vs R-Y, R-Y-RGDS. Blue: Cell attachment, Green line Cell spreading. Cell attachment test was carried out 1.0 × 10^4^ cells/well (3.0 × 10^4^ cells/cm^2^) cells in each well (*n* = 5) (Incubation time: 0).

The cell attachment and cell spreading activity of the peptide immobilized cell culture plate with NaCl adding system are shown in Figure 4. The cell adhesion of RGDS-R-Y was such that almost all the seeded cells adhered. In addition, the cell adhesion extension activity was significantly high. It is considered that the R-YRGDS has reduced cell attachment activity due to its weak peptide self-assembling. Since R-YRGDS has a weak self-assembling activity, it can be inferred that peptide dissolved in the medium which weakened the cell spreading activity. R-Y does not contain RGDS sequence in the peptide; it is considered that the cell attachment ability and cell spreading activity were lower than those of the RGDSR-Y. The R-RGD-Y also showed good results in cell attachment and cell spreading.

The above results summarize that the cells of integrin recognize the RGD sequence and the cell were able to attach to the peptide modified substrate.

## 3. Materials and Method

### 3.1. Materials

All 9-fluorenylmethyloxycarbonyl (Fmoc) amino acid derivatives and solid phase peptide synthesis resins were purchased from Watanabe Chemical Industries Ltd. (Hiroshima, Japan). All other reagents including organic solvents were purchased from FUJIFILM Wako Pure Chemical Corporation (Osaka, Japan).

### 3.2. Peptide Synthesis

We designed three types of barnacle proteins with self-assembling peptides in Table 1. R-Y related peptides that induce the cell aggregate were synthesized by the solid phase peptide synthesis procedure [49,50]. The R-Y and its related peptides were synthesized on Alko-PEG resin using a handmade standard manual Fmoc-protocol with a 4-(4,6-dimethoxy-1,3,5-triazin-2-yl)-4-methylmorpholinium chloride (DMT-MM) activation procedure [51,52]. N-α-(9-fluorenylmethoxycarbonyl)-L-proline-tritylcarboxamidomethyl polyethylene glycol resin (Fmoc-Tyr(tBu)-Alko-PEG-Resin (0.20 mmol) was placed in a polypropylene column (Column PD-10, Empty, Merck&amp Co. Inc, Rahway, NJ, USA) and washed three times with N, N-dimethylformamide (DMF) and methanol. The resin was swollen with 25% (*w*/*v*) dimethyl sulfoxide (DMSO)/DMF for 30 min. The mixture was then reacted with 20% (*w*/*v*) piperidine (PPD)/DMF for 30 min to deprotect the Fmoc group. Then, 0.6 mmol Fmoc-protecting amino acid, 0.06 mmol N-methylmorphiline, and 1.2 mmol DMT-MM were added to the resin. The condensation reaction was run for 120 min. The resin was washed six times with DMF for 1 min each time. The Fmoc group deprotection and condensation reactions were repeated to synthesize Arg(Pbf)-Arg(Pbf)-Lys(Boc)-Tyr(tBu)-Ser(tBu)-Gly-Ile-Leu-Gly-Asp(OtBu)-Leu-Ile-Gln(Trt)-Val-Ala-Val-Ile-Arg(Pbf)-Tyr(tBu)-Tyr(tBu)-Alko-PEG Resin. Finally, peptide protecting group deprotection and resin cleavage were conducted under acidic conditions. The protected peptide-Alko-PEG-Resin and the cleavage mixture (8.50 mL trifluoroacetic acid (TFA), 0.50 mL thioanisole, 0.80 mL pure water, 0.25 mL 1,2-ethanedithiol, and 0.75 g phenol) were stirred for 150 min [49,50]. Then, diethylether was added to reaction mixture, and the precipitate was filtered and dissolved in 50 mL of water. The crude R-Y peptide containing solution was dialyzed with a membrane with a molecular weight cutoff range of 100–500 Da (Spectra/Por Dialysis Membrane Biotech CE Tubing, MWCO: 100–500D). Other peptides were also synthesized in the same manner as above. Finally, all peptides were purified by high-performance liquid chromatography (HPLC 8020 System; Tosoh Corp., Tokyo, Column: TSKgel-ODS-100 V 5μm) with a gradient of water/acetonitrile containing 0.1% TFA (20–90% water/acetonitrile), (Flow: 1.0 mL/min, UV absorption wavelength: 210 nm, column oven temperature: 40 °C). All peptides were identified by matrix-assisted laser desorption/ionization mass spectrometry (MALDI-TOF-MS; Microflex LRF System, Bruker Corp., Billerica, MA, USA) using α-cyano-4-hydroxycinnamic acid as a matrix (Sigma-Aldrich Co. LLC, Saint Louis, MO, USA).

### 3.3. Secondary Structure Analysis by Infrared Absorption (IR) Spectrum

The FT-IR measurement was carried out by an FT/IR-4200 Fourier transform infrared spectrophotometer (FT-IR) (JASCO Corporation, Japan), and characterized amid band I [53]. The detector used was MCT (Hg1-xCdxTe) cooled with liquid nitrogen for 30 min. For the measurement, the wave number was 1600 to 1700 cm^−1^ and the number of integrations was 160 times. The peptide secondary structure was analyzed by multivariate analysis of the obtained spectrum (Spectra Manager Ver. 2, JASCO). All samples were obtained by freeze dry from 1 mg/mL peptide solution. The IR was measured in reflection mode using ATR (ATR Pro450-S, JASCO) in the solid state.

### 3.4. Secondary Structure Analysis by Circular Dichroism (CD) Spectrum

Secondary structure analysis was performed using a circular dichroism dispersometer (J-1500: JASCO Corporation, Japan). The peptide samples were prepared by mixing a 200 µM aqueous peptide solution and an aqueous NaCl solution so that the NaCl concentration was 0.0 to 0.1 M and the peptide concentration was 100 µM. The measurement was performed at 25 °C (measurement wavelength of 190 to 250 nm, a data interval of 1 nm, a scanning speed of 100 nm/min, a cell length of 0.1 cm, and 6 integrations). 

### 3.5. Preparation of Peptide Hydrogel and Evaluation of Physical Properties

A 2.0, 4.0, 6.0 wt% aqueous solutions, each containing one of R-Y, R-Y-RGDS, RGDS-R-Y, and R-RGD-Y peptides and a double concentration Dulbecco’s modified Eagle’s medium (DMEM: NISSUI PHARMACEUTICAL CO., LTD. Tokyo, Japan) (2 × DMEM) were prepared. (Main component of DMEM: NaCl; 6400 mg, KCl; 400 mg, CaCl_2_; 200 mg, glucose; 1000 mg, total of amino acid; 1056 mg/1.0 L) [54]. A hydrogel was prepared by mixing an aqueous peptide solution and 2 × DMEM in a volume ratio of 1: 1. Rheology measurements were performed using a rheometer to confirm the formation of hydrogels. HAAKE MARS 40 rheometer (Thermo Fisher Scientific Co., Ltd. Waltham, MA, USA) was used for rheology measurement, and a flat plate with a diameter of 8 mm was used. The hydrogel was then further prepared, placed in a silicon mold having a diameter of 8 mm, and allowed to stand at 37 °C under humidified conditions for 1 h. After that, the hydrogel was placed on the stage of the rheometer, and the measurement was performed under the conditions of 37 °C, frequency of 0.160 Hz, shear strain of 1%, and a measurement time of 10 min.

### 3.6. Observation of Microstructure of Peptide Hydrogel with Scanning Electron Microscope (SEM)

A hydrogel was prepared by the method shown in 2.5, and the hydrogel was dropped onto mica.

Then, chemical fixation was carried out with 4% paraformaldehyde/30 mM HEPES buffer for 2 h. After washing with 30 mM HEPES buffer, dehydration treatment was performed with a gradient system using ultrapure water, ethanol, and tert-butanol. Finally, an SEM sample was prepared by freeze-drying. SEM observation was performed with an Au vapor deposition thickness of 15 mm and an accelerating voltage of 10 kV.

#### 3.6.1. Cells and Culture

Mouse fibroblasts (L929) (RIKEN Biosource Research Center, Japan) were cultured in Dulbecco’s modified Eagle’s medium (DMEM: NISSUI PHARMACEUTICAL CO., LTD. Tokyo, Japan) containing 10% fetal bovine serum (FBS, HyClone; Cytiva, Sheffield, UK), 100 U/mL penicillin, and 100 μg/mL streptomycin (Invitrogen). All cells were maintained at 37 °C in a humidified 5% CO_2_/95% air atmosphere. Eagle’s Minimal Essential Medium (EMEM: NISSUI PHARMACEUTICAL CO., LTD. Tokyo, Japan) powder, 2.82 g was dissolved in 261 mL of ultra-pure water, sterilized in an autoclave at 120 °C for 15 min, and then filtered and sterilized. To prepare 10% (*w*/*v*) FBS EMEM medium, 7.50% sodium hydrogen carbonate (NaHCO_3_) aqueous solution 6.00 mL, 3% L-glutamine aqueous solution 3.00 mL, and 30 mL of FBS were added. In addition, 2.82 g of Eagle’s Minimal Essential Medium (EMEM) powder was dissolved in 291 mL of ultrapure water, sterilized in an autoclave at 120 °C for 15 min, and then filtered. To prepare serum-free EMEM medium 7.50%, NaHCO_3_ aqueous solution 6.00 mL, 3% L-glutamine, and 3.00 mL of aqueous solution were added.

#### 3.6.2. Preparation of Peptide-Immobilized Cell Culture Plate

A 200 μM aqueous solution and a 0.2 M NaCl aqueous solution were prepared with each peptide of R-Y, R-Y-RGDS, RGDS-R-Y, and R-RGD-Y. A 100 μM peptide aqueous solution (0.2 M NaCl) was prepared by mixing the peptide aqueous solution and the 0.2 M NaCl aqueous solution at a volume ratio of 1: 1. After the preparation, the mixture was added dropwise to a 96 well plate to a concentration of 80 μL/well. Then, it was dried under reduced pressure overnight at room temperature. 

#### 3.6.3. Cell Culture on a Peptide-Immobilized Substrate

The plate prepared in 3.6.2 was sterilized by UV irradiation for 1 h. The L929 with a passage number of 10 was seeded at 1.0 × 10^4^ cells/well (3.0 × 10^4^ cells/cm^2^, Incubation time; 0 h) and incubated for 6 h under 5% CO_2_ and 37 °C. After incubation, the unattached cells were removed from the peptide-immobilized substrate by washing PBS. In the meantime, an observation was performed using a phase-contrast microscope after 6 h. After culturing, the MTT solution was added dropwise to a concentration of 10 μL/well. After the dropwise addition, the mixture was incubated for 4 h under 5% CO_2_ and 37 °C. Then, the solubilizing reagent was added dropwise to 100 μL/well, and the mixture was incubated overnight at 5% CO_2_ and 37 °C. Finally, the absorbance was measured with a plate reader (TECAN Japan Co., Ltd. Kanagawa, Japan) at a measurement wavelength of 560 nm and a reference wavelength of 660 nm. Finally, percentage of cell attachment was calculated of based on seeded cells (Incubation time; 1 h).

## 4. Summary

The results of the CD spectrum confirm that the β-sheet structure of R-Y is dependent on the NaCl concentration. A similar tendency was observed for RGDS-R-Y and R-RGD-Y. However, it is suggested that the RGDS-R-Y contain more β-sheet structure than the R-Y. Since the R-RGD-Y showed a spectral pattern similar to the R-Y, it is considered that it has the same structure as the R-Y. On the other hand, the R-Y-RGDS has a random coil structure in aqueous solution, and β-sheet structure in NaCl solution. Similar results were obtained from the IR spectrum. The above secondary structure analysis clarified that changing the modification position of the RGDS affects the inherent secondary structure forming ability of the self-assembling peptide.

From the results of rheology measurement, with R-Y, RGDS-R-Y and R-RGD-Y were formed at 1, 2, and 3 wt%. With R-Y-RGDS, G’ and G” of 2 wt% and 3 wt% peptide hydrogels showed extremely small values. This correlates with the results of the secondary structure analysis and G’ of R-Y-RGDS, which has the weakest ability to form β-sheet structures. In R-Y-RGDS, G’ and G” of 2 wt% and 3 wt% peptide hydrogels showed extremely small values. This correlates with the results of secondary structure analysis, and G’ of R-Y-RGDS, which has the smallest ability to form β-sheet structures. It can be inferred that there is a correlation between the β-sheet structure forming ability and the mechanical strength of the hydrogel. When the cell attachment test was carried out by the peptide modified substrate, the cell activity was higher in self-assembling. It was suggested that changing the modification position of the RGDS on a self-assembling peptide affects the ability to form secondary structures of the peptide and the physical properties of the peptide hydrogel. When modifying a bioactive motif on a self-assembling peptide and designing a functional scaffold material, it is recommended that the modification position of the bioactive motif should be examined.

## Figures and Tables

**Table 1:** Peptide sequence of designed molecules.

Code	Amino Acid Sequences (One Letter Amino Acid Code)
R-Y (control)	RRKYSGILGDLIQVAVIRYY
R-Y-RGDS	RRKYSGILGDLIQVAVIRYYGRGDS
RGDS-R-	RGDSGRRKYSGILGDLIQVAVIRYY
R-RGD-Y	RRKYSGIRGDLIQVAVIRYY

**Table 2:** Results of MALDI-TOF MS spectra analysis and secondary structure analysis of synthetic peptide by FT-IR spectra.

Code	Date of MALDI-TOF-MS				% of Secondary Structure		
	Fw (Cal.)	[M]^+^ or [M+H]^+^	[M+Na]^+^	[M+K]^+^	β-Sheet	β-Turn	Other
R-Y (control)	2383.79	2383.9	-	-	67	16	17
R-Y-RGDS	2856.24	2853.2	-	-	50	25	25
RGDS-R-Y	2856.24	2858.7	2881.26	-	68	16	16
R-RGD-Y	2426.82	2424.7	-	-	64	17	19

**Table 3:** Results of G’ value of rheological analysis for various peptide concentration.

	R-Y (Pa)	R-Y-RGDS (Pa)	RGDS-R-Y (Pa)	R-RGD-Y (Pa)
1 wt%	290.9 (±18.4)	*^1^	179.5 (±12.2)	219.1 (±12.4)
2 wt%	912.2 (±49.9)	8.5 (±6.0)	990.2 (±60.1)	924.5 (±46.8)
3 wt%	3125.1 (±152.2)	3.8 (±2.7)	2444.2 (±15.8)	3872.1 (±21.4)

*^1^: sol state, G’ value of rheological analysis was average of *n* = 5.

## Data Availability

Not applicable.

## Supplementary Materials

The following are available online at https://www.mdpi.com/1422-0067/22/3/1240/s1, Figure S1. FT-IR spectra of synthetic R-Y derivative peptide.
