# Supplementary material for: Design of RGDS Peptide-Immobilized Self-Assembling β-Strand Peptide from Barnacle Protein
Source: Int J Mol Sci. 2021 Jan 27;22(3):1240. doi: 10.3390/ijms22031240 (PMC7866236; doi:10.3390/ijms22031240)

SI-Figure 1 FT-IR spectra of synthetic R-Y derivative peptide.

(A) R-Y, (B) R-Y-RGDS, (C) RGDS-R-Y, (D) R-RGD-Y.

Black line; Measurement data, Red line; α-Helix, Green line; β-sheet, Blue line; β-turn, Orange line; other.

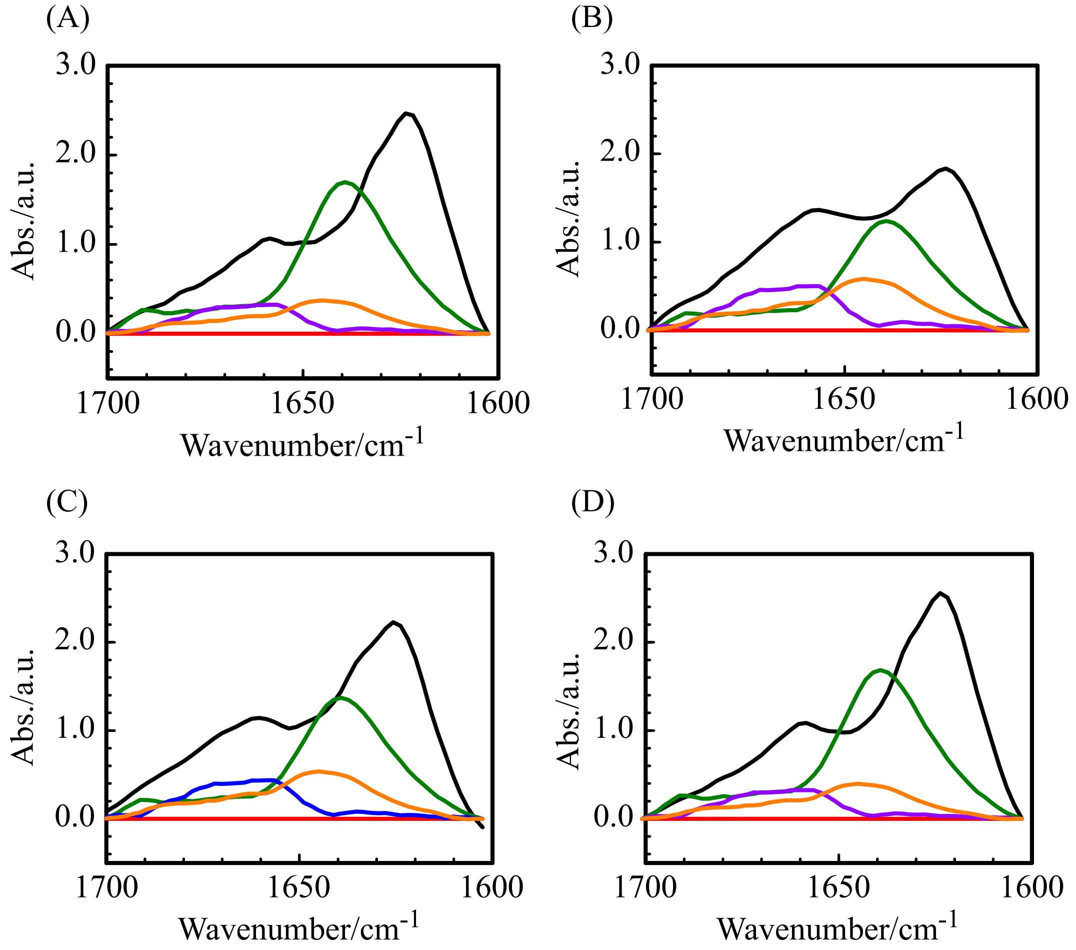

Supplement: Supplementary file 1 [file ijms-22-01240-s001.pdf]
